# Biomass distribution of sympatric mammals in a European temperate forest

**DOI:** 10.1098/rsos.250472

**Published:** 2025-08-06

**Authors:** Andro Pleskalt, Sebastião Farias, Toni Vicedo, Jose Manuel Álvarez-Martínez, José Valentín Roces-Díaz, Carlo Meloro, Soraia Pereira, Antonio Cruz, Jesús García, Pablo Gómez, María Ángel Lamillar, Elena Marsella, Manuel Jesús Varas, Elena Álvarez, Fredrik Dalerum

**Affiliations:** ^1^ Department of Biology, University of Zagreb, Zagreb, Croatia; ^2^ Department of Statistics and Operational Research, University of Lisbon, Lisbon, Portugal; ^3^ Biodiversity Research Institute, CSIC - University of Oviedo - Principality of Asturias, Mieres del Camino, Principality of Asturias, Spain; ^4^ Department of Biology of Organisms and Systems, University of Oviedo, Oviedo, Spain; ^5^ Research Centre in Evolutionary Anthropology and Palaeoecology, School of Biological and Environmental Sciences, Liverpool John Moores University, Liverpool, UK; ^6^ Imatec Innovación S.L., Colindres, Cantabria, Spain; ^7^ General Directorate of Biodiversity, Environment and Climate Change, Santander, Cantabria, Spain; ^8^ Department of Zoology, Stockholm University, Stockholm, Stockholm County, Sweden; ^9^ Mammal Research Institute, Department of Zoology and Entomology, University of Pretoria, Pretoria, Gauteng, South Africa

**Keywords:** biomass distribution, community ecology, trophic downgrading, temperate forest, camera trapping, occupancy models

## Abstract

The distribution of biomass among trophic levels and different types of organisms is a key characteristic of ecosystems. However, such biomass distributions might exhibit large perturbations owing to human activities. We used long-term camera trap data from a temperate forest ecosystem within the Cantabrian range, northern Spain, to quantify the biomass distribution of 10 mammal species ranging in body mass from 300 g to almost 100 kg. The species are representative of three distinct orders and include trophic levels from primary to secondary consumers. The observed biomass distribution was bottom-heavy with a disproportionally low biomass of secondary consumers, in particular large-bodied predators. In addition, the ratio of plant biomass to the biomass of mammalian primary consumers was over 6000 : 1 for total above-ground-plant biomass and over 700 : 1 for plant biomass available for mammal consumption. We suggest that the observed biomass distribution both among mammals and between mammals and plants provide an example of trophic downgrading, and highlight the radical effects human activities might have on the structure of terrestrial ecosystems.

## 1. Introduction

Even though land covers only approximately 30% of the Earth’s surface, 85% of the total global biomass is located in terrestrial environments [1]. The distribution of that mass is pyramid-shaped, with primary producers having the largest share, followed by primary and secondary consumers. Primary production is mainly regulated by precipitation and growth period [2], whereas the biomass of higher trophic levels is completely dependent on transfer rates from lower levels. Bar-On *et al*. [1] highlighted that the expected ratio of primary producers (plants) to consumers (herbivores) is approximately 12 : 1. This number agrees reasonably well with Lindeman [3], who proposed that, on average, approximately 10% of available energy is transferred from a lower to a higher trophic level. However, a more recent study by Perkins *et al*. [4] demonstrated that predator biomass scales with prey biomass with a three-quarters power exponent. This results in proportionately less predator biomass being supported as prey biomass increases.

Through their wide-ranging influence on all environments on Earth, humans have changed how biomass is distributed among organism groups and trophic levels. For instance, present wild mammal biomass is lower, but total mammal biomass is higher, than pre-human values [1,5]. In addition, areas covered by forest, which is dense in plant biomass, are being replaced by cropland and pastures. This has resulted in an overall decline in plant biomass on Earth [6,7]. On the other hand, human influences can also cause increased plant biomass in relation to higher trophic levels. These effects can be sufficiently strong to cause marine ecosystems, whose natural biomass distribution is top-heavy, to be transformed into a bottom-heavy biomass distribution [8].

The temperate zones are located between 23.5 and 66.5° latitude in the northern and southern hemispheres. The climate within these zones is characterized by mild mean temperatures and a distinct seasonality, both in temperature and precipitation. A wide range of ecosystems can be found in temperate areas, including temperate forests, temperate grasslands and even deserts [9]. Owing to its climate, the temperate zone is home to a significant portion of the world’s human population [10] and is the most productive in terms of agriculture [11]. Extended agriculture has caused a big destruction of temperate habitat [11,12], which combined with the large human population, has had negative effects on biodiversity in temperate areas [13,14].

Mammalia is a well-known class of animals that consists of approximately 6000 extant species [15]. Although mammals contribute a relatively limited amount to the total global consumer biomass [1], many species have disproportionally important roles in the functioning of terrestrial ecosystems [16,17]. For instance, small-bodied mammals are important pollinators and consumers of arthropods [16], while larger ones can have a significant impact on landscape modification [18]. Mammals also influence community structures of autotrophs, which in turn affect other vertebrates and invertebrates [16], as well as disease dynamics, wildfires, carbon capturing, invasive species and biochemical exchanges [19]. In addition, many mammals are important conservation targets as flagship and umbrella species [20].

We quantified the distribution of mammal biomass among trophic and taxonomic groups in a temperate European forest, which has seen long-term influences of human activity. We also compared the estimated values of mammalian herbivore biomass, i.e. primary consumers, to empirical estimates of vascular plant biomass. However, since much of the biomass in temperate forests is unavailable for herbivores, either by the trees being too tall or by the biomass being captured in non-edible vegetation types [21], we compared the estimated mammal biomass to both total above-ground biomass and to net primary productivity (NPP). NPP has been suggested as a good proxy for the amount of plant biomass that is available to herbivores [22]. We justify the comparison with total above-ground biomass by arguing that tall trees at some point must have escaped herbivory to grow. Hence, large accumulations of plant biomass not available for herbivores are an indication of limited herbivore pressure, albeit not in recent ecological times [23]. We specifically tested support for the following predictions: (i) biomass distribution will follow a 12 : 1 ratio of plants to primary consumers, i.e. mammalian herbivores; (ii) there will be a 90% loss of mammal biomass between primary to secondary consumers following a 10% biomass transfer between trophic levels; and (iii) mammal biomass distribution follows a three-quarters power exponent scaling between primary and secondary consumers. We base these expectations on proposed biomass ratios and transfer rates by Lindeman [3], Bar-On *et al*. [1] and Perkins *et al*. [4]. Although we appreciate these predictions are founded on theory developed for whole ecosystems, i.e. on average values across all organisms at a trophic level, we still regard them as informative as benchmarks even for studies on specific taxonomic groups. To test these predictions, we estimated the biomass of 10 mammal taxa: six from the order Carnivora, three from the order Artiodactyla and one from the order Rodentia. These species included four primary consumers, four secondary consumers and two omnivores feeding on multiple trophic levels.

## 2. Methods

### 2.1. Study area

**Figure 1.**
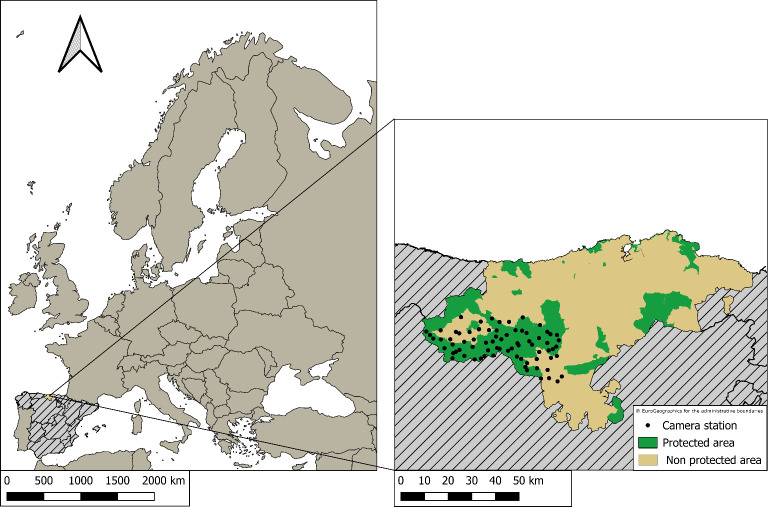
Location of the Autonomous Community of Cantabria in northern Spain as well as the locations of the trap camera stations within the province.

The study area is located in the province of Cantabria, northern Spain (figure 1). Most of it lies inside the protected areas Parque Nacional de los Picos de Europa, Parque Natural Saja-Besaya, Parque Natural Montaña Palentina and ZEPA Liébana and Parque Regional Montaña de Riaño y Mampodre. The study area covers 1125 km^2^ and stretches 130 km east to west and 70 km north to south. Cantabria has an average human population density of 109 individuals km^−2^, with most people living in and around the two cities of Santander and Torrelavega [24]. The topography of the study area is rugged, with elevations ranging from 400 to 2000 m.a.s.l. The climate is Atlantic with mild winters (minimum average temperature of 9°C) and relatively cool summers (maximum average temperature 20°C) [25]. Precipitation is approximately 1000 mm yr^−1^. Vegetation varies with elevation, ranging from pastures to deciduous forests, which are composed of beech (*Fagus sylvatica*), oak (*Quercus* sp.), holly (*Ilex aquifolium*) and birch (*Betula* sp.), as well as chestnut (*Castanea sativa*) and hazel (*Corylus avellana*) [26]. Mammals present in the region include large carnivores such as the grey wolf (*Canis lupus*) and the brown bear (*Ursus arctos*), a number of mesocarnivores (e.g. Eurasian badger, *Meles meles*; red fox *Vulpes vulpes*), large herbivores such as the red deer (*Cervus elaphus*), the roe deer (*Capreolus capreolus*) and the wild boar (*Sus scrofa*), as well as a number of smaller mammals [27].

### 2.2. Camera trapping

Camera trap data came from a survey commissioned by the Government of Cantabria and managed by the environmental consultancy IMATEC [28]. The survey was initiated in 2016 and consisted of cameras placed in a grid of 5 × 5 km cells. From 2016 until 2018, there were 36 cells, whereas 14 cells were added in 2019, for a total number of 45 cells from 2019 to 2022. One camera was placed randomly inside each cell, but the placements were constrained so that the minimum distance between two cameras was always above 3.5 km. The intent was to maintain the cameras in the same location throughout the study, but on 38 occasions, cameras were relocated within cells owing to theft or vandalism. This resulted in a total of 86 unique camera locations used for the study. There was never more than one camera active within a single cell at the same time. We, therefore, regard each cell to be a spatially independent sample unit. Each camera was placed on a tree trunk approximately 1 m above the ground. To draw the animal into the optimal position for camera trapping, a bait made of fish scent was placed approximately 7 m in front of each camera. Every camera was visited monthly for service, image downloads and reapplication of bait [28].

The survey used single motion-triggered digital cameras (Bushnell Trophy Cam Aggressor No Glow, Bushnell Corp., Overland Park, Kansas, USA). The angle of view of each camera was 35°, and the angle of detection was 43.9°. Maximum distance of detection during the day was 25 m and during the night 20 m. The trigger speed of each camera was 0.6 s. With each trigger, the camera took one photo and a brief video. During the day, the video was 30 s long and during the night 15 s. After each set of a photo and video, 6 s had to pass until the camera could be triggered again.

### 2.3. Estimation of abundance, density and total biomass of mammals

We extracted data from 7 October 2016 to 29 May 2022, resulting in a total of 79 896 camera days, with an average of 929 ( ± s.d. 241 days, min = 39, max = 1241) active days for each camera. We restricted the study to non-domestic or feral species with at least 400 individual observations to secure sufficient data for abundance modelling. Hence, although both dogs (*Canis familiaris*), domestic cats (*Felis catus*), sheep (*Ovis aries*), goats (*Capra hircus*) and cattle (*Bos taurus*) frequently occurred in the camera records, they were not included in our assessment of mammal biomass. We regarded independent observations of the same species to be those of different camera stations or at the same station but separated by at least 15 min. With this criteria, we selected 10 species for analyses: roe deer, red deer, wild boar, grey wolf, European wildcat (*Felis silvestris*), pine marten (*Martes martes*), Eurasian badger, brown bear, red fox and red squirrel (*Sciurus vulgaris*). We point out that this selection omitted some potentially important groups of mammals, most notably small rodents (Rodentia), shrews (Soricidae) and bats (Chiroptera). However, the omitted species were either very rare, as indicated by the lack of sufficient observations in the camera record for reliable abundance estimation, or would not have contributed to substantial amount of biomass owing to a combination of small body size and relatively low densities [27]. We therefore argue that we have captured the majority of the biomass of wild mammals with the included species. The number of observations per species varied from 408 for the brown bear to 9320 for the red fox, and the number of stations the species were observed at ranged from 53 (red squirrel) to 85 (wild boar, red fox and pine marten) (electronic supplementary material, table S1).

We used a Bayesian implementation of the single-season N-mixture model proposed by Royle [29] to estimate the relative abundance (average number of animals per camera station) of each species from the camera trapping data. This class of model is particularly useful to estimate abundance from unmarked individuals. The model was implemented using a Bayesian framework for the statistical environment R (http://r-project.org, v 4.4.2 for Linux) in the contributed package umbs (v 1.27, [30]). This package is based on the Stan language [31], which is using the No-U-Turn sampler for the Markov chain Monte Carlo process [32]. We selected a Bayesian implementation since they seem more robust to small sample sizes, and also provide abundance estimates with lower bias and higher precision than maximum likelihood implementations [33]. We fitted one single-season model for each species rather than using one of the many multi-species or multi-season extensions, since these either provided poor model fit or suffered from failure in the stabilization of the Markov process. We believe that these issues were owing to insufficient data for these more complex classes of models. However, to account for possible violation of population closure, we treated each camera and year as a separate sample unit, and added camera identity as an abundance level covariate [34]. This exchange of temporal to spatial dimensions may be justified if the key interest is abundance across the area, and not temporal change in abundance across time [35]. We fitted the models using observation data pooled over five-day periods to avoid an excess number of camera events without observations, but fitted the number of active camera nights within each five-day period as an observation level detection covariate. The models were fitted using a Poisson distribution with a log link for abundance and a binomial distribution with a logit link for detection. For each model, we ran four parallel chains of 4000 samples each and discarded the first 2000 iterations. Following Link & Eaton [36], we did not thin the remaining draws, hence yielding a final set of 8000 samples for estimating the average abundance of each species. Convergence of the Markov chains was assessed using trace plots and the Gelman-Rubin coefficient ([37]; electronic supplementary material, figure S1).

We converted the abundance estimates to densities by multiplying the estimated abundance of each species by the average group size of that species and then divided the resulting number of individuals by the median reported home range size for that species in similar environments (electronic supplementary material, table S1). We regard this as a sensible approximation of the estimated capture area for each camera and species. This area was set to be the same for all cameras. To calculate total abundance of a species, we multiplied the estimated density by the total area of the camera trapping grid (1125 km^2^). Finally, we estimated the biomass of each species by multiplying the total abundance by the species’ estimated adult body mass, retrieved from Spanish body mass data reported in Blanco ([27]; electronic supplementary material, table S1). Only adult body masses were taken. If there was a range of masses given, or exact masses of multiple individuals, an average was calculated. If there were masses of female and male individuals, again, the average of those was used.

### 2.4. Estimation of plant biomass

Total above-ground plant biomass was calculated using remote sensing technologies including Sentinel-2 imagery and Light Detection and Ranging (LiDAR) data though a combination of direct and indirect approaches [38]. We applied three statistical models based on Sentinel-2 imagery [39–41], and additionally used the FUSION software tool for LiDAR assessments on woody biomass [42]. Results were rasterized at a 10 m spatial resolution and averaged at the pixel level. Pixels were averaged to obtain tonnes ha^−1^ (areas of 10 × 10 pixels) and these values were aggregated to tonnes km^−2^, which were aggregated for each of the 5 km^2^ cells, which were finally aggregated to obtain an estimate of total biomass (electronic supplementary material, figure S2). As a heuristic accuracy assessment, the obtained biomass values were confirmed to agree with field data for the Cantabrian province obtained from the most recent version (4th) of the Spanish National Forest Inventory [43].

We used NPP as an index of plant biomass available for herbivore consumption. We used the MOD17A3HGF v061 product at a 500 m spatial resolution [44]. We downloaded data for our study areas for the period 2016−2022 and aggregated the values initially at the 5 km^2^ cells. We averaged the values for each cell across the whole study period, and then divided the temporal averages with estimated above-ground biomass for that cell. We then multiplied the average proportion of NPP in relation to total above-ground biomass calculated across all cells by the total estimated above-ground biomass across the study areas to achieve a measure of plant biomass available for mammal consumption. This was further used to calculate the average available biomass km^−2^. We note that the observed values of total above-ground biomass and NPP correspond to broad estimations for the study area, which we aggregated across scales by using different data sources of spatial information.

## 3. Results

Plant biomass in the whole study area was estimated to 9 367 452 tonnes for total above-ground biomass and 1 170 932 tonnes for biomass estimated as available for mammal consumption, corresponding to biomass densities of 8 326 tonnes km^−2^ and 1 041 tonnes km^−2^, respectively. The ratio of plant to mammalian primary consumers was approximately 6 300 : 1 for total above-ground plant biomass and 754 : 1 for plant biomass estimated as available for mammal consumption. Estimated biomass of the 10 mammal species was 1 548 tonnes in the whole study area, which corresponds to an average of 1.38 tonnes of mammals km^−2^. Within mammals there was a 3.7% biomass transfer from primary to secondary consumers (ratio 27 : 1). Primary consumers contributed to 95% of the total estimated mammal biomass (1 470 tonnes, or 1 307 kg km^−2^), followed by secondary consumers contributing to 4% of mammal biomass (55 tonnes, or 49 kg km^−2^) and omnivores contributing to 1% (23 tonnes, 20 kg km^−2^) (table 1). The order that contributed with most biomass was Artiodactyla with 95% (1 470 tonnes, or 1 306 kg km^−2^), followed by Carnivora with a contribution of 5% (78 tonnes, or 69 kg km^−2^). There was a very limited contribution of Rodentia, which contributed with less than 0.1% to mammal biomass (0.4 tonnes, or 0.38 kg km^−2^). The species with the highest average densities were roe and red deer, followed by the red fox and wild boar, whereas brown bear and wolf had the lowest average densities (table 1). Both roe deer and red deer also contributed with most biomass, followed by wild boar. Pine marten and red squirrel had the lowest contribution to mammal biomass (table 1).

**Table 1.** Total abundance (animals within the whole study area), animal density (individuals km^−2^), total biomass (tonnes within study area), biomass density (kg km^−2^), contribution of biomass of each species to the total estimated biomass (%) of 10 mammal species as well as primary producers in the Cantabrian range, northern Spain. Abundances of mammals were estimated from an N-mixture model of repeated species observations from camera trap photographs and biomass estimated by multiplying the densities by literature estimates of group sizes and body mass for each species. The biomass of primary producers was estimated both as the total above-ground biomass and as biomass available for mammal consumption. Both of of these values were estimated through remote sensing techniques. Bold values indicate biomass pooled across a trophic group.

trophic group / order	scientific name	common name	**abundance** **(animals in study area)**	**density** **(animals km^−^** ^**2**^ **)**	**biomass** **(tonnes in study area)**	**biomass** **(kg km^−^** ^**2**^ **)**	% of mammal biomass
**secondary consumers**					**55.04**	**48.92**	**3.6 %**
Carnivora	*Vulpes vulpes*	red fox	8 240	7.32	49.44	43.95	3.2 %
Carnivora	*Canis lupus*	wolf	115	0.10	3.44	3.06	0.2 %
Carnivora	*Felis silvestris*	European wildcat	313	0.28	1.33	1.18	0.1 %
Carnivora	*Martes martes*	pine marten	636	0.57	0.83	0.73	0.1 %
**omnivores**					**22.60**	**20.08**	**1.4 %**
Carnivora	*Meles meles*	Eurasian badger	1 780	1.58	21.36	18.98	1.3 %
Carnivora	*Ursus arctos*	brown bear	13	0.01	1.24	1.10	0.1 %
**primary consumers**					**1 469.89.00**	**1 306.57**	**95.0 %**
Arctiodactyla	*Cervus elaphus*	red deer	9 113	8.10	656.11	583.21	42.4 %
Arctiodactyla	*Capreolus capreolus*	roe deer	19 140	17.01	468.92	416.82	30.3 %
Arctiodactyla	*Sus scrofa*	wild boar	4 598	4.09	344.86	306.54	22.3 %
Rodentia	*Sciurus vulgaris*	red squirrel	1 412	1.26	0.42	0.38	<0.1 %
**primary producers^a^**					**9 367 452.00**	**8 326 620.00**	
available for mammal consumption^b^					1 170 932.00	1 041 828.00	

^a^ Total above ground biomass.

^b^ Estimated from average annual net primary production 2016−2022.

## 4. Discussion

The observed mammal biomass was biased towards primary consumers, but the biomass of mammalian primary consumers was very low in relation to that of plants, both for total above-ground biomass and for biomass estimated as available for mammal consumption. Furthermore, the observed biomass transfer rate between mammalian primary to secondary consumers was only one-third of the expected 10% transfer per trophic level proposed by Lindeman [3], and also substantially lower than the three-quarters power exponential transfer rate proposed by Perkins *et al*. [4]. Furthermore, herbivore biomass was exceptionally low in relation to plant biomass with an observed ratio of over 6000 : 1 for total above-ground biomass and over 700 : 1 for biomass estimated as available for mammal consumption. Even if we highlight that our values are not based on all organism groups across the trophic levels, we argue that these observations highlight a strong shift towards lower trophic levels in this temperate forest ecosystem, both within Mammalia and across trophic levels. Furthermore, our data mostly came from protected areas, which generally hold higher mammal abundance compared to unprotected land [45]. Hence, the estimated mammal abundances probably represent the upper end of the range of mammal biomass occurring in southern European temperate forests. We note that our results are in general agreement with both Serrano-Zulueta *et al*. [22], who found low baseline herbivory within Spanish protected areas, as well as global values proposed by Greenspoon *et al*. [5], who similarly to us found a particularly large contribution of Artiodactyla to the total biomass of all terrestrial mammals on Earth.

We suggest that the observed biomass ratios most likely were related to human modifications of this terrestrial ecosystem. These modifications may have been the result of several processes. For instance, humans may have altered the biomass distribution either by disproportionately targeting top-level consumers in eradication campaigns [19] or by hunting the largest herbivores for food [46]. The latter modification may have contributed to this region becoming forested, by allowing earlier succession stages to escape herbivory into current growth forms where large parts of plant biomass are not available for herbivore consumption [23]. In addition, other types of habitat modifications could also have caused a defaunation of this terrestrial ecosystem, leading to a bias towards plant biomass [47]. Habitat modifications may have been particularly important since they can have disproportionally large influences on large-bodied species and species that feed on high trophic levels [48].

The observed mammal biomass was higher compared to what has previously been observed in European temperate forests (0.74 tonnes km^−2^ [49], and in the tropical rain forest of Gabon, east Africa (1.00 tonnes km^−2^ [50]), but similar to the observed biomass of introduced mammals in New Zealand (2.09 tonnes km^−2^ [51]). In Gabon, most mammal biomass consisted of elephants (*Loxodonta africana cyclotis*) and primates. However, the ratio of prey to predator biomass was similar to the ones observed in our study (35 : 1 versus 27 : 1), suggesting similar trophic transfer rates. On the other hand, the savannah ecosystem of the Serengeti had a ratio of approximately 280 : 1 [52], although this low ratio of predators to prey may have been caused by high densities of migratory herbivores [53,54].

Our results indicated a disproportionally high depletion of secondary consumers in relation to primary consumers, and, in particular, a depletion in large-bodied predators. We note that this result was derived without including small mammals in our estimate, which are the primary prey for the majority of the secondary consumers included in the study. Such trophic downgrading has been observed elsewhere [19], and is supported by global mammal biomass estimates [5]. Since predation is an important ecological process [55], loss of large predators can have negative influence on ecosystem structure and function [56]. Hence, despite suggestions that large carnivores are recovering in European landscapes [57], our results highlight that European landscapes are heavily depleted in large-bodied predators and hence possibly disrupted by a lack of predation processes.

Although we based our study on a long-term dataset and well-established statistical methodology, we do offer some caveats to our results. First, both limited number of observations as well as a sub-optimal sampling design, with a fixed grid of 5 × 5 km for all species, could have caused low precision in the abundance estimate for some species. Second, the density estimates are very sensitive to the effective area around each camera. We used home range values recorded from literature to specify these areas. However, although these literature values may have added uncertainty to the biomass estimates, we used home range sizes recorded from the same study area or from as similar environments as possible. Furthermore, we have assumed that the whole study area was saturated with animals. This may not have been accurate, at least not for all species. For instance, a species such as the roe deer is primarily confined to the borders of forests and fields, and, therefore, has relatively small home ranges. This may result in inflated total abundance estimates if the densities are extrapolated across larger regions. However, despite these methodological concerns, we argue that our results are at least qualitatively robust.

To conclude, the observed biomass distribution of 10 mammal species in a European temperate forest suggested a strong trophic downgrading. The distribution of mammalian biomass was biased towards lower trophic levels with a disproportionally high biomass of primary consumers. However, the biomass ratio of primary producers to mammalian primary consumers was exceptionally high, even if plant biomass not available for consumption was excluded. We argue that our study represents a strong example of ecological changes, quite possibly caused by human activities such as hunting and land transformation. We suggest that the depletion of mammals and, in particular, species feeding at higher trophic levels may have profound ecological as well as evolutionary consequences.

## Data Availability

Formatted data of mammal observations, R-code for the abundance analyses and spatial data for plant biomass estimation is available from Figshare [58]. Supplementary material is available online [59].
